# Exploring the potential of community health workers in type-2 diabetes and hypertension management in Cambodia

**DOI:** 10.1371/journal.pone.0351958

**Published:** 2026-06-23

**Authors:** Savina Chham, Por Ir, Josefien Van Olmen, Veerle Buffel, Ngovlily Sok, Vannith Hay, Willem van de Put, Wim Van Damme, Edwin Wouters

**Affiliations:** 1 National Institute of Public Health, Phnom Penh, Cambodia; 2 Centre for Population, Family and Health, Department of Social Sciences, University of Antwerp, Antwerp, Belgium; 3 Department of Family Medicine and Population Health (FAMPOP), Faculty of Medicine and Health Sciences, University of Antwerp, Antwerp, Belgium; 4 Department of Sociology, Vrije Universiteit Brussel, Brussels, Belgium; 5 Department of Public Health, Institute of Tropical Medicine, Antwerp, Belgium; 6 Department of Gerontology, Faculty of Medicine and Pharmacy, Vrije Universiteit Brussel, Brussels, Belgium; 7 Centre for Health Systems Research and Development, University of the Free State, Bloemfontein, South Africa; No institution, UNITED KINGDOM OF GREAT BRITAIN AND NORTHERN IRELAND

## Abstract

The burden of type-2 diabetes (T2D) and hypertension (HTN) in Cambodia is a major concern. The government and donors have introduced several interventions, yet human resource shortages hamper their implementation. Community health workers (CHWs) can be a valuable additional resource for health in T2D and HTN management. The current study aimed to assess (1) CHWs’ knowledge, attitude, and practices (KAP) in T2D and HTN management and (2) the potential health system barriers for incorporating CHWs in T2D and HTN management. This mixed-method study comprised a survey among 153 active CHWs to assess their KAP towards T2D and HTN, and semi-structured interviews with key informants were conducted to understand the challenges of the health system and propose solutions in incorporating CHWs in T2D and HTN management. Approximately 90% of CHWs correctly answered general knowledge questions on NCDs, risk factors, and prevention; however, only around 20–40% correctly answered questions on family history or tobacco use as risk factors. Most respondents appeared to have positive attitudes and have been practicing some activities related to T2D and HTN. Both financial and non-financial resource constraints were cited as challenges of the health system; therefore, re-structuring the definition of CHWs’ roles and responsibilities, and assessing the need and workload have been proposed as ways forward to effectively incorporate CHWs in T2D and HTN care. CHWs in Cambodia have shown their potential in T2D and HTN management; however, a well-designed strategy, including technical training, clearly defined roles and responsibilities, and strong support structure, is important to maximize their potential in the health system.

## Introduction

Cambodia has shown advanced progress in achieving the Millennium Development Goals (MDGs) for maternal and child health (MCH) and infectious diseases [1]. Yet, the burden of non-communicable diseases (NCDs) such as type-2 diabetes (T2D) and hypertension (HTN) is a major concern for the country. A 2016 STEPS survey estimated the prevalence of T2D and HTN among adults aged 40–69 years in Cambodia as 3.3% and 23.5%, respectively [2].

The Royal Government of Cambodia’s strong political commitment towards achieving Universal Health Coverage (UHC) provides the Ministry of Health (MoH) with an opportunity to improve health service delivery [3]. To ensure service availability for T2D and HTN, the MoH and donor organisations have introduced a multitude of primary healthcare (PHC) interventions, including (1) establishment of Non-Communicable Disease (NCD) clinics at referral hospitals (RHs), (2) introduction of the World Health Organization Package of Essential Non-Communicable Disease Interventions (WHO PEN) programme in health centres (HCs), and (3) expansion and integration of MoPoTsyo’s community-based Peer Educator Network [4].

Ensuring universal access to T2D and HTN healthcare services through scale-up of health intervention packages is one of the stepping stones towards UHC for NCDs in Cambodia; however, it has been severely constrained by health system challenges such as weak governance, limited financial resources, and especially, a lack of human resources [5,6]. Human resources are needed for T2D and HTN service delivery at the PHC and community levels since managing these NCDs require a comprehensive and cross-cutting approach—including life-long healthcare support, early case detection, psychosocial promotion, self-management, and medical support [7].

To adequately address the growing need for T2D and HTN services in the context of the PHC system struggling due to limited human resources for health, community health workers (CHWs) have increasingly been recognised as a promising frontline healthcare workforce to support the PHC system additionally [8,9]. The Alma Ata Declaration of 1978 established CHWs as an essential element of the comprehensive PHC approach—“suitably trained socially and technically to work as a health team and respond to the community’s expressed health needs” [10]. Increasingly, CHWs are being seen as a prerequisite for the health system to achieve the goal of UHC [11] through various tasks performed in different health programmes. Previous studies from other low- and middle-income countries (LMICs) have demonstrated that CHW-based programmes are effective in HTN and diabetes control, reducing coronary heart disease risk [12,13], helping improve blood pressure [14], and are beneficial in diabetes management [15]. A study in Pakistan showed that family-based home education delivered by CHWs achieved effective lowering of blood pressure levels in the population [16]. With insufficient numbers of trained health professionals at the PHC level, CHWs could thus also be a valuable extension of the professional healthcare team in NCD health services in Cambodia and a crucial step in scaling up management of NCDs.

In Cambodia, CHW programmes are a part of the broader national “Community Participation Policy for Health” [17]. In this policy, CHWs are referred to as “Village Health Support Group” (VHSG) members and are volunteers at HCs [18]. Apart from VHSGs, CHWs are known by many different names due to their engagement in specific health interventions implemented at the PHC level by various Vertical Health Programmes (such as HIV/AIDS, infectious diseases (Tuberculosis or Malaria) and MCH [19–21]) in which they have extra roles. However, so far, engagement of CHWs in T2D and HTN healthcare services has been limited in the country.

Since Cambodia’s strategic action plan to address the NCD epidemic in a sustainable manner is to have both facility-based and community-based interventions [22], assessing the competences of CHWs to contribute to NCD management is crucial to evaluate their potential in national efforts to scale-up interventions that address the growing burden of T2D and HTN. Although some other countries such as Uganda [23] and South Africa [24,25] have studied CHWs’ knowledge on NCDs, this information was lacking in Cambodia. In response to this knowledge gap, our study sought to provide an overview of CHWs’ knowledge, attitude, and practices (KAP) in T2D and HTN management. In addition, it was also necessary to assess whether the health system is ready and willing to incorporate CHWs as a central tool in its care for T2D and HTN. We thus also sought to assess the potential health system barriers for incorporating CHWs in T2D and HTN management.

## Methods

### Study design and data collection

The study was conducted in six operational districts (ODs) spread across different provinces in Cambodia: OD Sotr Nikum (Siem Reap), OD Daunkeo (Takeo), OD Samrong (Oddormeanchey), OD Kong Pisei (Kampong Speu), OD Pearaing (Prey Veng), and OD Sen Monorom (Mundulkiri). These ODs were purposively selected reflecting the different T2D and HTN health system interventions at the PHC level—the selection details of the first five ODs are well-described in previous publications [4,26]. However, OD Sen Monorom was additionally selected to provide extra value to the study due to the existing Malaria Vertical Programme. The research design of this study comprised (1) a cross-sectional survey among CHWs and (2) semi-structured interviews with key informants (KIs). The purpose and procedure of the study was explained to all participants and their participation in the study was voluntary. The personal information of the participants was kept confidential. Written informed consent was obtained from all study participants and/or their legal guardians prior to conducting the study. The research has been performed in accordance with the Declaration of Helsinki and the protocol was approved by the National Ethics Committee for Health Research (No. 295/NECHR on 25 November 2019) in Cambodia.

The cross-sectional survey was conducted using a multi-stage cluster sampling method where six ODs were purposively selected first. Then, 75 villages were randomly selected, and exhaustive sampling was used to invite all available CHWs from the selected villages to participate in the survey since the number of active CHWs per village varied due to different reasons. As a result, 153 CHWs participated in the study among 160 invited (96% response rate). A structured questionnaire was used in the local language (Khmer) and included questions on: (a) socio-demographic characteristics, (b) characteristics of CHWs, and (c) the knowledge, attitude, and practices (KAP) in T2D and HTN management (S1 File).

a. Socio-demographic variables included age, gender, marital status, level of education, main occupation, and annual income.
b. Characteristics of CHWs included number of titles for their roles in providing community work, duration (years) of working as a CHW, duration (hours) per month spent on CHW work, and number of households under their supervision.
c. The KAP section for CHWs on T2D and HTN included seven questions on general NCDs, five questions on risk factors of T2D, five questions on prevention of T2D, five questions on risk factors of HTN, five questions on prevention of HTN, four questions on attitude, and two questions on practice.

The development of the questionnaire was guided by an extensive review of relevant literature, government guidelines [22,27], and previous publications [28,29], along with consultations with health system and NCD experts from the study team. This questionnaire was contextualised to overcome biases as a result of different education and literacy levels. To ensure validity, the questionnaire was pre-tested among CHWs at the study settings to ensure local acceptability and clarity.

The survey was conducted at the CHWs’ homes or any convenient place determined by CHWs themselves. After receiving consent from each CHW, socio-demographic data and characteristics of the CHW were collected through an interview-based questionnaire. For the KAP section, the data collector read out questions one at a time (in the order that they appeared in the questionnaire) and asked whether the participant thought the statement was true or false. The participant could only respond “Yes” to any question they thought was true and “No” to any question they thought was false. The knowledge score was calculated by assigning one point to each correct answer. The same scoring method was applied to questions on attitudes and practices. All interviews were conducted in the local language by well-trained data collectors.

Semi-structured interviews with KIs were conducted to understand their in-depth perspectives on the potential roles of CHWs in the health programme, including T2D and HTN management, the challenges of the health system, and their proposed solutions in incorporating CHWs in T2D and HTN management (S2 File). There were a total of eleven KIs: five were policymakers (two from the departments of the MoH (KI01 and KI02), and three from the Vertical Health Programmes (HIV/AIDS, Malaria, and Tuberculosis; KI03–KI05)) and six were implementers (representatives from ODs; KI06–KI11). The interviews were conducted by two researchers (SVC asked the questions and NSL/HVN took notes) in the local language and in the KIs’ respective offices, and notes were taken as an interview record note in the local language. These notes were translated into English by two research assistants who were familiar with qualitative methods. The study was conducted from December 2019 to April 2020. Confidentiality of the notes was maintained by the research team throughout the data collection, analysis, and publication. We followed an iterative cycle to determine data saturation, in which data analysis occurred concurrently with data collection. The research team simultaneously conducted interviews and SVC developed the coding. When no new themes emerged from the interviews, the team agreed that data saturation had been achieved.

### Data analyses

Survey data were managed and analysed using STATA version 14 (StataCorp. 2015. Stata Statistical Software: Release 14. College Station, TX) and included generation of frequencies and percentages using descriptive statistics.

All the semi-structured interviews were read by the first author (SVC) to immerse herself in the data. An inductive approach was used to allow themes to be identified and studied in detail by using NVivo software version 12 plus (NVivo qualitative data analysis software; QSR International Pty Ltd.). Discussions were carried out with the research team to have a final development of themes that were in line with the study objectives.

## Results

### Socio-demographic variables and characteristics of CHWs

Table 1 presents the socio-demographic variables and characteristics of the CHWs. Among all participants, the mean age was 49 years (standard deviation; SD = 14), 51.0% were females, 79.1% were currently married, 43.8% had at least completed primary education, 39.2% were farmers, and 48.4% had an annual income of 4–17 million riels (USD 1000–4250).

**Table 1:** Socio-demographic variables and characteristics of study participants (N = 153).

Socio-Demographic Variables and Characteristics (N = 153)	Mean ± SD (range) or N (%)
**Age (years)**	49.0 ± 14.0 (17–81)
**Age Group**	
≤ 39 years old	41 (26.8%)
≥ 40 years old	112 (73.2%)
**Gender**	
Male	75 (49.0%)
Female	78 (51.0%)
**Marital Status**	
Currently married^1^	121 (79.1%)
Single	7 (4.6%)
Divorced or widowed	25 (16.3%)
**Level of Education** ^**2**^	
No education or less than primary school	53 (34.6%)
At least complete primary school	67 (43.8%)
Complete secondary or higher	33 (21.6%)
**Main Occupation**	
None/stay at home	10 (6.5%)
Farmer	60 (39.2%)
Vendor	23 (15.0%)
Chief/vice-chief/member of commune council	52 (34.0%)
Other^3^	8 (5.2%)
**Annual Personal Income**	
less than 1 million riels (<USD 250)	10 (6.5%)
1–4 million riels (USD 250–1000)	58 (37.9%)
4–17 million riels (USD 1000–4250)	74 (48.4%)
More than 17 million riels (> USD 4250)	11 (7.2%)
**Number of roles performed** (community health-related work)	
Only one role	68 (44.5%)
Two roles	47 (30.7%)
Three roles	38 (24.8%)
**Main roles of the CHW in the health programme**	
Health Centre-Village Health Support Group	121 (79.1%)
Tuberculosis Vertical Programme (Community Direct Observation Therapy Watchers)	23 (15.0%)
Other (Village Malaria Worker or Mobile Malaria Workers, Peer Educator for T2D and HTN, Coordinator for social protection, Community-based Distributor for Contraceptive Pill)	9 (5.9%)
**Average no. of years as a CHW**	8.6 ± 6.5 (0–31)
**Average CHW work hours (per month)**	17.0 ± 17.0 (2–96)
**Average no. of households under supervision**	148.0 ± 97.0 (17–492)

^1^Currently married included long-term married.

^2^If an individual did not start school or started primary school but did not complete Grade 6 of Cambodian education system, their educational attainment is classified as “no education or less than primary school”. If an individual finished primary school, but did not finished secondary school, their educational is classified as “at least complete primary school”. If an individual finished secondary school or higher education, we classified them as “complete secondary and higher”.

^3^“Other” referred to any occupation that did not fall into four listed categories such as factory workers etc.

The CHWs had spent on average 8.6 years (SD = 6.5) as a CHW. In our study, 44.5% of respondents performed only one role, 30.5% performed two roles, and 24.8% performed three roles linked to community health-related work.

The current roles included (1) being a formal VHSG who is involved in community out-reach activity of the HCs, (2) observing whether tuberculosis patients take their medicines as Community Direct Observation Therapy Watchers, and other roles such as (3) diagnosing and treating malaria as Village Malaria Workers (VMWs) or Mobile Malaria Workers (MMWs), (4) peer educator for T2D and HTN, (5) Red Cross volunteers, (6) working for non-government organisations’ social protection programmes, and (7) distributing contraceptive pills as Community-Based Distributors.

### Knowledge, attitude, and practice

As presented in Table 2, most respondents answered about six out of seven NCD-related knowledge items correctly (M = 6.3, SD = 0.8). The majority of CHWs (90.2%) agreed that NCDs cannot directly spread between people, 96.7% agreed that NCDs can be prevented with a healthy diet, 91.5% agreed that diabetes is an NCD, 94.8% did not agree that regular exercise will put you at risk of diabetes, 93.5% agreed that HTN is an NCD, and 94.8% agreed that reducing salt intake may reduce the risk of high blood pressure. The highest prevalence of misunderstanding was seen in item 7 (related to smoking) of NCD-related knowledge, where 74.5% incorrectly believed the statement was true.

**Table 2:** Response to knowledge, attitude and practice items.

#	Knowledge, Attitude and Practice items	Correct (%)
	**NCD-related knowledge**	
1	Do you think non-communicable disease is one that cannot be directly spread between people?	90.2
2	Do you think non-communicable disease can be prevented by having healthy diet?	96.7
3	Do you think of diabetes as a non-communicable disease?	91.5
4	Do you think doing regular exercise will put you at risk of having diabetes?	94.8
5	Do you think hypertension is a non-communicable disease?	93.5
6	Do you think reducing salt intake may reduce risk of having high blood pressure?	94.8
7	Smoking is not a risk factor for non-communicable diseases.	74.5
	**Type-2 Diabetes Risk factor**	
1	Family history of T2D is a risk factor of having T2D.	23.5
2	Fruit and vegetable intakes are not risk factors of T2D.	88.9
3	Lack of exercise is a risk factor of T2D.	86.9
4	Tobacco use is a risk factor of T2D.	48.4
5	Walking exercise is not a risk factor of T2D.	96.1
	**Type-2 Diabetes Prevention**	
1	Do exercise regularly can prevent you from having T2D.	96.7
2	Having too much of oily food cannot prevent you from having T2D.	89.5
3	Vegetable consumption can prevent you from having T2D.	92.2
4	Sweet beverages cannot prevent you from having T2D.	92.8
5	Physical activities can prevent you from having T2D.	94.1
	**HTN risk factor**	
1	Salty food is a risk factor of having HTN.	96.7
2	Fruit and vegetable intakes are not risk factors of HTN.	93.5
3	Lack of exercise is a risk factor of HTN.	95.4
4	Tobacco use is a risk factor of HTN.	70.6
5	Walking exercise is not a risk factor of HTN.	96.7
	**HTN prevention**	
1	Do exercise regularly can prevent you from having HTN.	96.7
2	Having too much of oily food cannot prevent you from having HTN.	86.3
3	Vegetable consumption can prevent you from having HTN.	94.1
4	Eating salty food cannot prevent you from having HTN.	94.1
5	Physical activities can prevent you from having HTN.	96.1
	**Attitudes**	
1	If you ever heard about diabetes, do you think it is important for patients to receive on-time treatment?	98.7
2	If you ever heard about diabetes, do people with diabetes need lifelong treatment?	84.9
3	If you know something about high blood pressure/hypertension, do you think that it is important for people over 40 years old to have their blood pressure measured regularly?	99.3
4	If you know something about high blood pressure/hypertension, do people with high blood pressure/hypertension need to take medication regularly?	99.3
	**Practices**	
1	Have you ever advised people over 40 years old in this village to have their blood glucose checked (measured) at the health facility?	83.0
2	Have you ever advised people over 40 years old in this village to have their blood pressure measured?	85.6

On average, about three out of five T2D risk-factor knowledge items were answered correctly (M = 3.4, SD = 0.9). The majority of CHWs agreed that lack of exercise (86.9%) is risk factor of T2D, while fruit and vegetable intake (88.9%) and walking exercise (96.1%) are not risk factors of T2D. A minority of participating CHWs correctly agreed that a family history of T2D (23.5%), and tobacco use (48.4%) are risk factors of T2D.

On an average, the participating CHWs scored 4.6 out of 5 on the T2D prevention knowledge items (M = 4.6, SD = 0.7). The CHWs acknowledged that ways of preventing T2D include regular exercise (96.7%), not having too much oily food (89.5%), vegetables consumption (92.2%), not drinking sweet beverages (92.8%), and doing physical activities (94.1%).

Similarly, around four out of five HTN risk-factor knowledge items were answered correctly (M = 4.5, SD = 0.6). The CHWs acknowledged that risk factors of HTN include eating salty foods (96.7%), lack of exercise (95.4%), tobacco use (70.6%), while walking exercise (96.7%), and fruit and vegetable intake (93.5%) are not a risk factor of HTN.

Lastly, approximately four of five HTN prevention knowledge items were answered correctly (M = 4.6, SD = 0.8). The CHWs acknowledged that ways of preventing HTN include exercising (96.7%), vegetables consumption (94.1%) and doing physical activities (96.1%). They agreed that having too much of oily food (86.3%) and eating salty food (94.1%) are not ways of preventing HTN.

Most respondents had a positive attitude on the importance of receiving on-time treatment for T2D patients (98.7%), the importance of lifelong treatment for T2D (84.9%), the importance for people over 40 years of age to receive HTN screening regularly (99.3%), and the importance of taking medicine regularly for HTN patients (99.3%). Since they had encountered some patients living with T2D and HTN in their villages, CHWs tended to be very positive on the statement on disease management including screening and treatment. Moreover, some CHWs had been informed about the T2D and HTN service availability at the HC, which allowed them to be able to inform and encourage villagers to have their blood pressure and blood glucose screened.

Regarding practices, 83.0% CHWs had ever advised people over 40 years of age to have blood glucose screening and 85.6% had ever advised those over 40 years of age to undergo blood pressure screening.

### Health system barriers and ways forward for incorporating CHWs in T2D and HTN management

Respondents reported on barriers of the health system related to the CHWs’ involvement in NCD management and on potential ways to address the reported barriers and the feasibility of implementation.

#### Health system barriers.

The major health system barriers reported were the need for both (1) financial and (2) non-financial support. Respondents recognised that other vertical disease control programmes also provide financial incentives (in the form of monthly payments, case-based incentives, transportation, or mobile phone top-up), and recognized this as an essential part of any community-based intervention. They also admitted that the limited government structural support increased the dependency on external donor funding and hindered the sustainability of CHW engagement.

*“Financial incentive is important. What we learnt from the malaria vertical programme was that this programme had a large funding and always succeeded in achieving its indicators; however, the shortage of funding in 2016 reduced the performance of all the programme activities including the VMWs. Why? Because there was no longer any amount of money to support the VMWs in their work, so they stopped working too. Therefore, I still agree that we need some money for them to work for us.”* (Representative from implementers-KI08)

*“Financial support is important even if CHWs are volunteers since they also have a family to support. CHWs are motivated to work if they can receive some financial incentive. To date, we can allocate some fund from our facility’s funding revenue (user-fees) which is around 5000 to 10000* [riels; USD 1.15 to 2.5] *only to compensate their transportation when they have to come to HC for a meeting. To fully operate their performance in the community for any healthcare programme, I think financial support is essential.”* (Representative from implementers-KI11)

Since there are no national Vertical Health Programmes supporting the CHWs for T2D and HTN management, public health facilities—especially HCs—have used their own financial revenues through user-fees to only support transportation fees for CHWs to attend meetings at the HCs. However, respondents stated that the current revenue from user-fees is low and limited to contribute as an incentive for CHWs’ work.

*“For sustainable financial support to CHWs, we cannot rely only on the revenues of user-fees of the HC since the amount is already low and it cannot be allocated much to support the CHWs’ work. I think government grant or funding is the most sustainable funding stream and the health sector reform through Decentralization and De-concentration would be an opportunity to engage local authorities. This reform meant the local authorities must have accountability to the health service management in their regions meaning they need to consider local resource allocation for the health-related activities in the communities.”* (Representative from the policymakers-KI03)

Respondents raised the fact that non-financial support such as training, supervision, and resource supply are also crucial components to ensure sustainability of the CHW programme and an empowerment on their performance. System structural support was raised as a complementary point to financial support; CHWs need proper training to enrich their skills and gain confidence. Respondents suggested that CHWs must receive pre-service and refresher training along with sufficient resource supplies.

*“For our vertical disease programme, we were able to achieve all the indicators including community function. CHWs have been involved since the start of the programme and received both financial and technical support including monthly incentive and material supply. We also offered them regular supervision to monitor and guide their performance. We provided them training and always conduct a regular monthly follow-up meeting for them to share work progress and challenges they encountered in the field. Our programme believed that this support is essential for us or another related health programme to achieve the outcome.”* (Representative from policymakers-KI05)

#### Ways forward to involve CHWs in the scale-up of NCD management.

The current employment system of CHWs in the country seems to be diverse and different from one region to another, based on the availability of national Vertical Health Programmes. The poorly-organised system structure, and undefined roles and responsibilities of CHWs seem to be barriers to their performance for T2D and HTN management at the PHC level. Respondents raised a need for re-defining the roles and responsibilities of CHWs to help them in receiving appropriate support for their roles that would increase their motivation and work performance leading to better implementation of health programmes.

*“I think that what constrains CHWs in receiving the financial support was how they were defined in our national policy as “a volunteer”. As they have been defined and labelled as volunteers for HCs and the community, it limited their odds of receiving some incentive or recognition as an important part of the PHC. This definition that labelled them as “a volunteer” should be changed so that they can be incentivised through their performance. We cannot expect anyone to work for free.”* (Representative from policymakers-KI04)

Respondents also noted the need for assessing the workload and competences of CHWs to ensure optimal performance. This should be done on a regular basis to deeply understand their performance, work challenges, and difficulties, which would help in the successful scale-up of CHW interventions through appropriate task assignment, structural support, and re-adjusted interventions to fulfil their need and address their challenges.

While CHWs have long been funded through separate national Vertical Health Programmes, respondents noted the need to rearrange the employment and financing system of CHWs in general. Scale-up of CHW interventions does not only entail the addition of new disease management interventions (like T2D and HTN) to their task, but also a rethinking of their competences, workload, and remuneration.

*“I think it is feasible to use the existing VHSGs in NCDs management; however, we need to understand their workload. With our programme financially supporting them, they still were programme-driven, and more concerned about their personal businesses, which posed as barriers to their performance. They tended to work on programmes that generated more funding for them.”* (Representative from policymakers-KI01)

*“A community-based intervention such as home-based care for people living with HIV/AIDS was a key activity in our Vertical Health Programme. Due to the shortage of funding, we wished to integrate our community intervention as one community function with another Vertical Health Programme. Since integration could save more funding when it is decreased. This community functions should be managed by one separated department where the department offers all the healthcare training to CHWs and manages the pool funding to support them in any health-related activities.”* (Representative from policymakers-KI03)

## Discussion

Our study provides evidence on the potential contribution of CHWs to scale-up T2D and HTN care in Cambodia by assessing their KAP as well as the health system barriers and potential solutions for successful implementation. Our main findings highlight the knowledge of CHWs in Cambodia, and identify key facilitators to CHW scale-up, namely strengthened support (both financial and non-financial) to CHWs and revised financing structures.

Overall, the general knowledge among CHWs on T2D and HTN was good, albeit still limited on certain risk factors. The latter finding is consistent with studies in other low-income settings such as in Eastern Uganda [29]. This might be explained by the fact that current training opportunities mainly consist of occasional information sharing, workshops from the HCs, and media advertisements on curative aspects with little attention to risk factors. A large proportion of CHWs were aware of the most common NCDs and the majority knew about prevention of T2D and HTN, reflecting their fundamental knowledge and the potential role of CHWs to engage in this programme. The demonstrated positive attitudes and good practices with regard to T2D and HTN potentially resulted from the high prevalence of T2D and HTN in their communities, as also indicated in the study in Uganda [29].

Despite assessing the CHWs’ KAP, it is also essential to understand the health system barriers that could hinder their performance in the future engagement in T2D and HTN health intervention. Our study found that the major barriers for further implementation of CHW interventions related to training, support, and financing. Capacity building was cited as an important step. To deepen and broaden their capacity in prevention and disease management, not only for T2D and HTN, but also related to connections with other diseases, CHW trainings should cover more disease areas, and include risk factors and preventive measures. A study in LMICs has also confirmed that the design of a targeted training curriculum was one of the key factors that could affect the effectiveness of a CHW training programme [30].

However, their performance is further affected by health system factors, more specifically resource constraints (financial and non-financial barriers). Substantial financial investment from the government is needed through an innovative funding mechanism to improve structural support. Previous studies have demonstrated that boosted tax revenues for NCDs could be a potential fund-raising mechanism; for instance, a study from Tonga has shown that the NCD tax policy can be successfully employed as a strategy to support health goals for NCD services [31]. The Thai Health Promotion Foundation has also been able to generate an annual revenue of USD 120 million from a 2% surcharge of excise tax on tobacco and alcohol [32], which was used to support evidence generation, a public campaign, and social mobilization to address NCD risk factors. Evidently, village health volunteers in Thailand are a key part of the PHC receiving 1000 Thai Baht (USD 32) per month [33]. In Cambodia, the government has issued sub-decree number 193 on 4 December 2019 on assigning health management and health service delivery to the capital and province administration [34]. This allows room for financial allocation of the provincial administrator budget to operate health related-activities [35]. The local governance must be more accountable to its population’s health through improved access to NCD care at public health facilities. This can be achieved by bringing services closer to the villagers and re-allocating local funds to NCD services including CHW engagement in all health services of the HC. A study that analysed the cost-effectiveness of CHWs in Mozambique showed that including a minimum wage for CHWs could lead to a large gain in efficiency in the implementation of health interventions [36].

Non-financial resources, such as continuous training, material support, or supervision from healthcare professionals were also cited as essential elements for CHWs’ engagement in T2D and HTN care. Previous studies have pointed out that the essential elements for an effective work environment for CHWs include supplies and equipment, supportive supervision, and respect from the community [23,37]. Inadequate supply could impact the community’s trust on the role of CHWs as has also been demonstrated in previous studies concerning CHW programmes [38,39].

The majority of the CHWs in our study had more than two roles including MCH or infectious diseases; therefore, over-burdening CHWs is a clear concern, which has also been indicated in other developing countries [40]. A previous study conducted in Cambodia has also outlined that CHWs had a number of roles and responsibilities as part of their volunteer work [41]. Therefore, the design of future community-based interventions should be planned rigorously to ensure adequate support to maximise CHWs’ performance and to avoid over-burdening. This is in line with a review on the influence of the work environment on CHWs’ productivity and effectiveness, which stated that a manageable workload carried out in an organised manner, with a reasonable geographic distance to cover, the needed supplies and equipment, a supportive supervisor, and respect and acceptance from the community and the health system are prerequisites for an effective community-based strategy [37].

CHWs can play a role as crucial extensions of the health workforce, especially in the scale-up of T2D and HTN care because they can provide services to the community such as raising awareness and improving screening, and can contribute to increasing coverage, addressing the horizontal dimension of scale-up—as defined in the previous multi-country research study [42]. Their special role in providing self-management support to patients can also contribute to expand the existing package of interventions through vertical scale-up.

Further research is needed on identifying the appropriate scale-up mechanisms for a comprehensive and effective community-based health intervention as a part of T2D and HTN management.

## Limitations and strengths of the study

Our study has some limitations especially related to its purposive selection of the six ODs, which restricts the generalisability of study findings to other areas of the country, and the limited sample size, which undermines the power of an in-depth analysis of quantitative data. Yet, the strength of our study was the high response rate from the CHWs and that it involved the synergies between both quantitative and qualitative data that enabled the triangulation of findings. The findings were not only obtained from CHWs but also from respondents from the Vertical Health Programmes who have long-term experience in engaging CHWs in their respective programmes. Interviews were documented using interview record notes rather than audio recordings due to participant-related constraints. This approach may have limited the verbatim capture of responses and possibly affected the depth of qualitative data; however, measures such as contemporaneous note-taking and review by the research assistant team specialised in qualitative research were used to reduce potential information loss.

## Conclusion

The evidence generated from our study will be beneficial as a stepping stone for future policy design on community participation as a part of T2D and HTN management in the Cambodian healthcare system.

The potential of CHWs in T2D and HTN management with their fundamental background could be maximised through well-designed community-based strategies that focus on their work characteristic, and support needed in the health system. The first strategy is to develop a technical support mechanism through a well-designed training curriculum that covers diverse topics to fill in their knowledge gaps, especially on risk factors. Second, their roles and responsibilities should be sharpened to their characteristics and be clearly defined to avoid over-burdening. A strong support structure is essential to ensure that the two strategies mentioned above can be fully and effectively implemented, thereby maximizing the potential of CHWs’ performance.

## Supporting information

S1 File Survey questionnaire. (DOC)

S2 File Questionnaire for key informant interview. (DOC)

## Abbreviations

CHW: Community Health Worker
HC: Health Centre
HTN: Hypertension
KAP: Knowledge, Attitude and Practices
KI: Key Informant
LMICs: Low- and Middle-Income Countries
MCH: Maternal and Child Health
MDGs: Millennium Development Goals
MMW: Mobile Malaria Worker
MoH: Ministry of Health
NCD: Non-Communicable Disease
OD: Operational District
PHC: Primary Healthcare
RH: Referral Hospital
SD: standard deviation
T2D: Type-2 Diabetes
UHC: Universal Health Coverage
VHSGs: Village Health Support Groups
VMW: Village Malaria Worker
WHO PEN: World Health Organization Package of Essential Non-Communicable Disease Interventions
