# Supplementary material for: Exploring the potential of community health workers in type-2 diabetes and hypertension management in Cambodia
Source: PLoS One. 2026 Jun 23;21(6):e0351958. doi: 10.1371/journal.pone.0351958 (PMC13289884; doi:10.1371/journal.pone.0351958)
Supplement: S1 File — (DOC) [file pone.0351958.s001.doc]

**Exploring the potential of CHWs in T2D and HTN management**

**Survey Questionnaire**

| **Section 1: Identification of Interview** | | |
| --- | --- | --- |
| Q1 | Operational District |  |
| Q1a | Administrative District |  |
| Q1b | Commune | :name___________________ code:[_________] |
| Q1c | Village | :name___________________ code:[_________] |
| Q1d | Health Center | :name___________________ code:[_________] |
| Q1e | Interviewer Code  *Example A1 (group A team leader)* | :[ ][ ] |
| Q1f | Name of Community Health Worker  *(Common name called by most of villagers)* | : text |
| Q1g | Code of Community Health Worker  *Example​ K01A1C01*  *Please put the code in consent form* |  |

| **Section 2:** **Socio-Demographic and Economic** | | |
| --- | --- | --- |
| **N** | **Question** | **Answer** |
| Q2 | How old are you? (write down age) | __________(years) |
| Q3 | What is your **gender**? (record male or female as observed) | 1.Male  2.Female |
| Q4 | Religion (one answer) | 1. Buddhist  2. Islam  3. Christian  4. Other: please specify……………… |
| Q5 | Marital Status (single answer) | 1. Currently married  2. Single  3. Divorced  4. Widowed  88.Refused |
| Q6 | What is the **highest level of school** you have completed?  (single answer) | 1. No formal schooling  2. Less than Primary school  3. Completed Primary School  4. Incompleted Secondary School  5. Completed Secondary School  6. Incompleted High School  7. Completed High School  8. Incompleted University  9. Completed University  10. Incompleted Post graduate  11. Completed Post graduate  88. Refused |
| Q7 | What is your **main occupation**?  (beside Community Health Worker) | 1. None/stay at home  2. Farmer  3. Vendor/Market seller  4. Chief of Village  5. Vice Chief of Village  6. Member of Commune Council  7. Retired  8. Labor worker  9. Other: please specify……  88. Refused |
| Q8 | How much in average is your **annual**  **income**? | 1. less than or equal 1 000 000 riels  2. More than 1 000 000 to less than 4 000 000 riels  3. More than 4 000 000 to less than 17 000 000 riels  4. More than 17 000 000 riels to less than 21 000 000 riels  5. More than 21 000 000 riels  88. Don’t know or Refused |
| **Section 3:Community Health Workers’ work characteristics** | | |
| Q9 | What are the titles you have in your work as a community health​ work? (select all apply)  (If more than 1 answer, go to Q10) | 1.Volunteer Health Support Group(VHSG)  2. Village Malaria Worker(VMW)  3. Mobile Malaria Worker(MMW)  4. Peer Educator for diabetes or hypertension  5. Peer Educator for HIV/AIDS  6.Red Cross Volunteers  7.Community Based Distributors  8.Community Home Based Care Teams  9.Community Direct Observation of Therapy Watchers  10.Mother Support Groups  11. Other: please specify…… |
| Q10 | If more than one title, which title do spend most of your time on? (only one answer) | 1.Volunteer Health Support Group  2. Village Malaria Worker  3. Mobile Malaria Worker  4. Peer Educator for diabetes or hypertension  5. Peer Educator for HIV/AIDS  6.Red Cross Volunteers  7.Community Based Distributors  8.Community Home Based Care Teams  9.Community Direct Observation of Therapy Watchers  10.Mother Support Groups  11. Other: please specify…… |
| Q11 | What exactly do you do as a community health worker?  ***(more than one answer)***  ***Please specify in additional records*** | Write down in heading paper (with code) |
| Q12 | How long have you been working as community health worker in this village?  *(Record the duration of the action since the first role to date)* | ___________(Years)  write 88 if refuse |
| Q13 | How many hours do you spent for all role that you have per month? | ___________(Hours)  write 88 if refuse |
| Q14 | How many households are you responsible for? | ___________(Households)  write 88 if refuse |
| Q15 | What are the reasons that you decide to be Community Health Worker?  (more than one answer)  15a. Help community health  15b. To receive training on healthcare  15c. To receive health information  15d. To gain more political power  15e. To receive free healthcare use (NSSF)  15f. To gain recognition from villagers  15g. Having family member or friends who worked as CHW as well  15h.Other: please specify …………. | 15a. 1.Yes 2.NO  15b. 1.Yes 2.NO  15c. 1.Yes 2.NO  15d. 1.Yes 2.NO  15e. 1.Yes 2.NO  15f. 1.Yes 2.NO  15g. 1.Yes 2.NO |
| Q16 | How satisfied are you with your current activities?  (Only one answer) | | 1. Very satisfied | | --- | | 2. satisfied | | 3. Don’t know | | 4. dissatisfied | | 5. Very dissatisfied | |
| Q17 | What are the barriers you face doing your work as community health worker?  (more than one answer)  17a.Time  17b. Family  17c. Case incentives  17d. Other incentives beside case  17e. Community Acknowledgement  17f. Capacity building on further learning opportunities  17g. Technical assistance  17h. Confidence to perform tasks  17i. Other, please specify ……. | 17a. 1. Yes 2. No  17b. 1. Yes 2. No  17c. 1. Yes 2. No  17d. 1. Yes 2. No  17e. 1. Yes 2. No  17f. 1. Yes 2. No  17g. 1. Yes 2. No  17h. 1. Yes 2. No |
| **Section** 4: Support (Materials, Training and Supervision) | | |
| Q18 | Have you received the materials to perform your work?  If “No” or “reject” or “don’t know”, please skip to Q20 | 1.Yes  2.No  88.Don’t know/ Refuse |
| Q19 | Do you think that the provided materials are enough for you to do your work? | 1.Yes  2.No  88.Don’t know/ Refuse |
| Q20 | Have you received training to perform your work?  If “No” or “reject” or “don’t know”, please skip to Q23 | 1.Yes  2.No  88.Don’t know/ Refuse |
| Q21 | How many times that you receive the training? | _____________ times  88. don’t know/refuse |
| Q22 | Is the training you received enough for you? | 1.Yes  2.No  88.Don’t know/ Refuse |
| Q23 | Do you receive financial support to perform your work?  If “No” or “reject” or “don’t know”, please skip to Q27 | 1.Yes  2.No  88.Don’t know/ Refuse |
| Q24 | If you receive, which form of financial support have you been receiving?  (more than one answer ) | 1.Monthly Salary  2.Case incentive (per diem)  3.Transportation fee  4.Mobile phone fee  5.Other, please specify ……… |
| Q25 | If you receive financial support, how much per month? | ____________________Riels per month  write 88 if refuse |
| Q26 | How much are you satisfied with your financial support? (Only one answer) | 1. Very dissatisfied  2.Dissatisfied  3.Neutral  4.Satisfied  5. Very satisfied   |  | | --- | |
| Q27 | Beside training, have you received technical support from your supervisor?  *(Technical support is like regular mentoring or consultation)*  If “No” or “reject” or “don’t know”, please skip to Q31 | 1.Yes  2.No  88.Don’t know/ Refuse |
| Q28 | Where is your supervisor (provide supervision and technical support) based?  *(only one answer)* | 1.Operational district  2.Health Center  3.National Program of the Ministry of Health  4.Non-Government Organization  5. Local authorities  6.Other, please specify ……….. |
| Q29 | How much are you satisfied with the support of your supervisor? | 1. Very dissatisfied  2.Dissatisfied  3.Neutral  4.Satisfied  5. Very satisfied |
| Q30 | How much are you satisfied the relationship of you with your supervisor? | 1. Very dissatisfied  2.Dissatisfied  3.Neutral  4.Satisfied  5. Very satisfied |
| Q31 | How much are you satisfied the relationship of you with healthcare worker? | 1. Very dissatisfied  2.Dissatisfied  3.Neutral  4.Satisfied  5. Very satisfied |
| Q32 | How much are you satisfied the relationship of you with villagers in your community? | 1. Very dissatisfied  2.Dissatisfied  3.Neutral  4.Satisfied  5. Very satisfied |
| Q33 | How much are you satisfied the relationship of you with other community health worker? | 1. Very dissatisfied  2.Dissatisfied  3.Neutral  4.Satisfied  5. Very satisfied |

| **Section 5:** Knowledge, attitudes and practices of NCDs  *I would like to ask some questions related to NCD, we will read each question and we want to know if they are correct.* | | |
| --- | --- | --- |
| Q34 | Do you think non-communicable disease is one that cannot be directly spread between people? | 1.Yes  2.No |
| Q35 | Do you think non-communicable diseases can be prevented by having a healthy diet? | 1.Yes  2.No |
| Q36 | Do you think of diabetes as a non-communicable disease? | 1.Yes  2.No |
| Q37 | Do you think doing enough exercise will put you at risk of having diabetes? | 1.Yes  2.No |
| Q38 | Do you think hypertension is a non-communicable disease? | 1.Yes  2.No |
| Q39 | Do you think reducing salt intake may reduce your risk of having high blood pressure? | 1.Yes  2.No |
| Q40 | Smoking is not a risk factor for non-communicable disease? | 1.Yes  2.No |
| *We would like to ask some more questions regarding diabetes* | | |
| Q41 | Have you ever heard about diabetes? | 1.Yes  2.No |
| Q42 | Which of the following factor are the risk factors of diabetes?  42a. Family history of diabetes  42b. Fruit and vegetable intakes are risk factors of T2D  42c. Lack of exercise is a risk factor of T2D  42d. Tobacco use is risk factor of T2D  42e. Walking exercise is risk factor of T2D | 42a. 1. Yes 2. No  42b. 1. Yes 2. No  42c. 1. Yes 2. No  42d. 1. Yes 2. No  42e. 1. Yes 2. No |
| Q43 | Which of the following practices can prevent you from having diabetes?  43a. Do exercise regularly can prevent you from having T2D  43b. Having too much of oily food can prevent you from having T2D  43c. Eating vegetable can prevent you from having T2D  43d. Sweet beverages can prevent you from having T2D  43e. Physical activities can prevent you from having T2D | 43a. 1. Yes 2. No  43b. 1. Yes 2. No  43c. 1. Yes 2. No  43d. 1. Yes 2. No  43e. 1. Yes 2. No |
| Q44 | If you ever heard about diabetes, do you think it is important for patients to receive on-time treatment? | 1.Yes  2.No  88.Don’t Know |
| Q45 | If you ever heard about diabetes, Do people with diabetes need lifelong treatment? | 1.Yes  2.No  88.Don’t Know |
| Q46 | Have you ever advised people over 40 years old in this village to have their blood glucose checked (measured) at the health facility? | 1.Yes  2.No  88.Don’t Know |
| Q47 | Do you think that you have enough information on diabetes to share to people in your village? | 1.Yes  2.No  88.Don’t Know |
| Q48 | Would you like to have more information or engage in diabetes activity? | 1.Yes  2.No  88.Don’t Know |
| *We would like to ask some more questions regarding hypertension* | | |
| Q49 | Have you ever heard about hypertension? | 1.Yes  2.No |
| Q50 | Which of the following factor are the risk factors of hypertension?  50a. Salty food is a risk factor of having HTN  50b. Fruit and vegetable intakes  50c. Lack of exercise  50d. Tobacco use  50e. Walking exercise | 50a. 1. Yes 2. No  50b. 1. Yes 2. No  50c. 1. Yes 2. No  50d. 1. Yes 2. No  50e. 1. Yes 2. No |
| Q51 | Which of the following practices can prevent you from having hypertension?  51a. Do exercise regularly  51b. Having too much of oily food  51c. Eating vegetable  51d. Eating salty food  51e. Physical activities | 51a. 1. Yes 2. No  51b. 1. Yes 2. No  51c. 1. Yes 2. No  51d. 1. Yes 2. No  51e. 1. Yes 2. No |
| Q52 | If you know something about high blood pressure/hypertension, Do you think that it is important for people over 40 years old to have their blood pressure measured regularly? | 1.Yes  2.No  88.Don’t Know |
| Q53 | Do people with high blood pressure/hypertension need to take medication regularly? | 1.Yes  2.No  88.Don’t Know |
| Q54 | Have you ever advised people over 40 years old in this village to have their blood pressure measured? | 1.Yes  2.No  88.Don’t Know |
| Q55 | Do you think that you have enough information on hypertension to share to people in your village? | 1.Yes  2.No  88.Don’t Know |
| Q56 | Would you like to have more information or engage in hypertension activity? | 1.Yes  2.No  88.Don’t Know |

| **Section 6: Self-motivation**  *If I read the following statement referring to you, to what extent do you agree that it really is something that reflects you* | | |
| --- | --- | --- |
| Q57 | My role as a CHW makes you feel good about yourself | 1.Strongly agree  2. Agree  3. Neutral  4. Disagree  5. Strongly disagree |
| Q58 | I am proud to work for the community by being a community health worker in my village | 1.Strongly agree  2. Agree  3. Neutral  4. Disagree  5. Strongly disagree |
| Q59 | I always complete your tasks efficiently | 1.Strongly agree  2. Agree  3. Neutral  4. Disagree  5. Strongly disagree |
| Q60 | I feel motivated to work as hard as a community health worker | 1.Strongly agree  2. Agree  3. Neutral  4. Disagree  5. Strongly disagree |
| Q61 | I am satisfied with the opportunity to use your abilities in your job as a community health worker in my villages | 1.Strongly agree  2. Agree  3. Neutral  4. Disagree  5. Strongly disagree |

| **Section 7: Participation in the NCD Program** | | |
| --- | --- | --- |
| Q62 | If you were given extra work to help with diabetes and hypertension program, would you like to accept additional role? | 1. Yes, it is acceptable  2. Not, I cannot accept it  88. Don’t Know |
| Q63 | If you accept additional role, how confident are you in working on diabetes and hypertension? | 1.Very confident  2. Confident  3.Neutral  4.Not confident  5.Not really confident |
| Q64 | How many days per week will you be willing to commit to work on diabetes and hypertension? | ______ Days (not more than 7 days)  88. Don’t know |
| Q65 | What would be the barriers for you to perform this extra work on diabetes and hypertension?  (Write in paper) | Recorded in code header |
| Q66 | What would be the facilitators for you to perform this extra work on non-communicable disease?  (Write in paper) | Recorded in code header |
| Q67 | Do you think it is important for people having diabetes or hypertension to have a good self-management? (only one answer)  (self-management for diabetes and hypertension including but not limited to regular medication taking, regular blood pressure or blood glucose checked up, consume healthy meal such as vegetables, fruits exercise and regular follow-up meeting with healthcare worker) | 1.Yes, very important  2. Important  3. Neutral  4. Not important  5. Not important at all |
| Q68 | Do you think how importance of the following person for providing support in self-management of diabetes and/or hypertension patient?  (one answer)  68a. Family  68b. Healthcare workers (doctors, nurses)  68c. Community health worker | 1.Yes, very important  2. Important  3. Neutral  4. Not important  5. Not important at all  1 2 3 4 5  1 2 3 4 5  1 2 3 4 5 |
| Q69 | As a community health worker, are you willing to support them in self-management? | 1. Yes 2. No   88. don’t know |
| Q70 | If you think you can help in self-management, which of the following activities you can involve in?  70a.Home visit  70b.Healthy lifestyle education  70c. Healthy diet education  70d.Health follow-up  70e.Reminder or guide to use medicinal  70f. Reminder on follow-up schedule  70g.Group training on self-management  70h. Individual training on self-management  70i.Other, please specify …………  record 88, if don’t know | 70a. 1. Yes 2. No  70b. 1. Yes 2. No  70c. 1. Yes 2. No  70d. 1. Yes 2. No  70e. 1. Yes 2. No  70f. 1. Yes 2. No  70g. 1. Yes 2. No  70h. 1. Yes 2. No |
| Q71. Before we conclude our survey, we would like to know that we can contact you again in the future for further interview?   If yes, please provide us your contact details  ** No | | |
| *That concludes out survey. Thank you for your time and before I leave, do you have any questions, comments or concerns?* | | |
|  | | |
| Interviewer/supervisor notes: Use this space to record notes about the interview with this participant, such as incomplete individual interview forms, number of attempts to re-visit, etc. | | |
|  | | |
