# Supplementary material for: Exploring the potential of community health workers in type-2 diabetes and hypertension management in Cambodia
Source: PLoS One. 2026 Jun 23;21(6):e0351958. doi: 10.1371/journal.pone.0351958 (PMC13289884; doi:10.1371/journal.pone.0351958)
Supplement: S2 File — (DOC) [file pone.0351958.s002.doc]

**Questionnaire for key informant interview**

**Protocol title**

Exploring the potential of community health workers in type-2 diabetes and hypertension management in Cambodia.

**Background**

Hello my name is………. I am a researcher at National Institute Public Health (NIPH). I would like to invite you to take part in my research study on exploring the potential of community health workers (CHW) in type-2 diabetes (T2D) and hypertension (HTN) management in Cambodia.

**The main objective of this study is to explore** the potential of CHWs in T2D and HTN management in Cambodia through conducting a survey on CHW to assess their knowledge, attitude and practice, and assessing the health system barriers and the proposed solutions from key stakeholders who are working in relation to any community-based intervention in Cambodia.

**Study procedures**

Our researcher will explain the study to you and the interview will be conducted in Khmer by a trained researcher. Key Informant interview, you will be asked of questions about your knowledge, opinion and solution of CHW role in T2D and HTN management in Cambodia.

You will be given time to read and understand the information provided in the information sheet and consent form. At that time, you are welcome to ask any questions about the study and we will clarify any doubts that you might have. You can choose to take part in the study after you have understood the purpose and procedure of the study. In addition, you may stop at any time during the survey and if there are questions that you would prefer not to answer, you do not have to.

The interview will last for at least 60 minutes.

| **N** | **Topic** | **Direction for Interview**  Start asking key informants about their professional experience. |
| --- | --- | --- |
| 1 | Knowledge | 1. Have you ever had any knowledge or experience of existing CHWs (program and region-based), their current roles and practice of CHWs in health care or health service delivery?   Prob: try to ask about village health support group and other CHW of vertical program.   1. Have you ever had any knowledge of potential CHW roles in health care and T2D and HTN program? How do you perceive their potential in those health interventions?   Prob: perception for those who are key informants who have previously engaged CHW in their own health program.   1. If you have any experience with CHW, can you share us how they were supported in term of supporting structure, formulation, engagement and challenges of engaging CHWs in vertical and horizontal program? |
| 2 | Opinion | 1. Could you share the best practice for integrating CHW into T2D and HTN program?   Prob: For those key informants from vertical program, try to ask for a story telling about their experience from one phase to another phase on having CHW in their program.   1. What were the barriers or facilitators you encountered in engaging CHW in your program? What could you expect as the barriers and facilitators in incorporate CHWs into T2D and HTN program. |
| 3 | Solution | 1. From your experience, what could be the solution to avoid burdening the CHW’s role or engagement in T2D and HTN management? What should the health system prepare to incorporate them in T2D and HTN management? 2. From your experience, what could be the solution to address the barriers and/or strengthen the facilitators of their future engagement in T2D and HTN management? 3. Have you identified or could you think of any sustainable financial mechanism for community-based intervention? |
